# Supplementary material for: Characterization of amyloid β fibril formation under microgravity conditions
Source: NPJ Microgravity. 2020 Jun 12;6:17. doi: 10.1038/s41526-020-0107-y (PMC7293247; doi:10.1038/s41526-020-0107-y)
Supplement: Supplementary file 2 — Supplemental Materials [file 41526_2020_107_MOESM2_ESM.pdf]

Supplmentary Figure 1

(A)

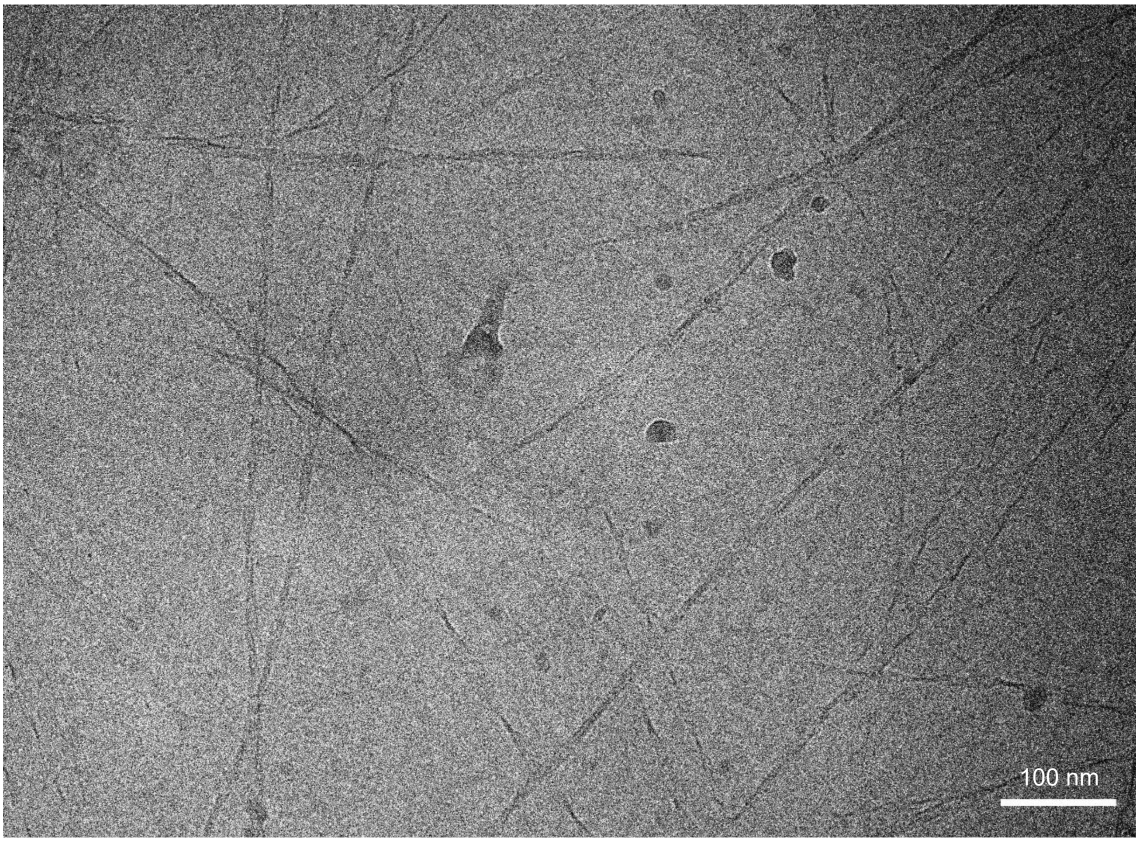

(B)

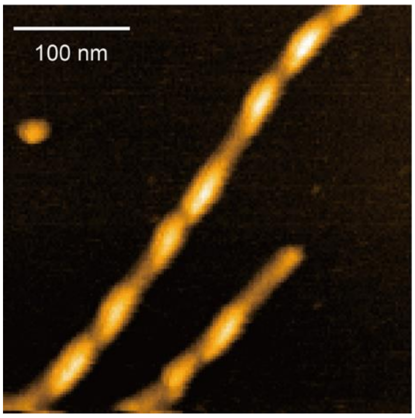

Supplementary Figure 1 (A) An overview cryo-EM image and (B) a typical AFM image of the Aβ(1-40) amyloid fibrils prepared under the microgravity condition. AFM images of amyloid fibrils placed on a freshly cleaved mica were acquired in the tapping mode at room temperature.
